# Environmental Design Strategies to Decrease the Risk of Nosocomial Infection in Medical Buildings Using a Hybrid MCDM Model

**DOI:** 10.1155/2021/5534607

**Published:** 2021-06-15

**Authors:** Lei Xiong, Ge Sheng, Zi-Mu Fan, Hua Yang, Feng-Jang Hwang, Bo-Wei Zhu

**Affiliations:** ^1^School of Architecture and Applied Art, Guangzhou Academy of Fine Arts, Guangzhou 511400, China; ^2^Faculty of Humanities and Arts, Macau University of Science and Technology, Macau 999078, China; ^3^Mural and Complex Material Painting Department, Hubei Institute of Fine Arts, Wuhan 430205, China; ^4^School of Mathematical and Physical Sciences, University of Technology Sydney, Sydney, Australia

## Abstract

The prevention and control of nosocomial infection (NI) are becoming increasingly difficult, and its mechanism is becoming increasingly complex. A globally aging population means that an increasing proportion of patients have a susceptible constitution, and the frequent occurrence of severe infectious diseases has also led to an increase in the cost of prevention and control of NI. Medical buildings' spatial environment design for the prevention of NI has been a hot subject of considerable research, but few previous studies have summarized the design criteria for a medical building environment to control the risk of NI. Thus, there is no suitable evaluation framework to determine whether the spatial environment of a medical building is capable of inhibiting the spread of NI. In the context of the global spread of COVID-19, it is necessary to evaluate the performance of the existing medical building environment in terms of inhibiting the spread of NI and to verify current environmental improvement strategies for the efficient and rational use of resources. This study determines the key design elements for the spatial environment of medical buildings, constructs an evaluation framework using exploratory factor analysis, verifies the complex dominant influence relationship, and prioritizes criteria in the evaluation framework using the decision-making trial and evaluation laboratory- (DEMATEL-) based analytical network process (ANP) (DANP). Using representative real cases, this study uses the technique for order preference by similarity to ideal solution (TOPSIS) to evaluate and analyze the performance with the aspiration level of reducing the NI risk. A continuous and systematic transformation design strategy for these real cases is proposed. The main contributions of this study include the following: (1) it creates a systematic framework that allows hospital decision-makers to evaluate the spatial environment of medical buildings; (2) it provides a reference for making design decisions to improve the current situation using the results of a performance evaluation; (3) it draws an influential network relation map (INRM) and the training of influence weights (IWs) for criteria. The sources of practical problems can be identified by the proposed evaluation framework, and the corresponding strategy can be proposed to avoid the waste of resources for the prevention of epidemics.

## 1. Introduction

Nosocomial Infection (NI) is a nonprimary infection that is contracted during hospital stay. It is also known as healthcare-acquired infection [1]. In China and the United States, the annual number of NI-related deaths is 1.25–3.5 million [2], and the direct economic loss due to Nosocomial Infection is about US$28–33 billion per year [3]. Nosocomial Infection increases the burden on the public health system and increases the medical risk and recovery time for patients. The prevention and control of Nosocomial Infection is becoming more difficult and the occurrence mechanism is becoming more complex. The abuse of antibiotics in many countries has led to an increase in multidrug resistance for bacteria, so the prevention and treatment of Nosocomial Infection is more difficult [4]. A globally aging population has also led to a continuous increase in the proportion of patients with a susceptible constitution [5]. The complex treatment process increases the risk of exposure to Nosocomial Infection and the frequent occurrence of severe infectious diseases increases the cost of the prevention and control of Nosocomial Infection [6]. In terms of SARS and COVID-19, Nosocomial Infection is the main mode of contagious infection in the early stage, so there are a large number of infections among medical staff. Between January 1, 2020, and January 28, 2020, 138 patients who were diagnosed with New Coronavirus pneumonia in Zhongnan Hospital in Wuhan University. It is estimated that about 41% of these infections were Nosocomial Infections and 29% involved medical staff.

In terms of architectural design, reducing the risk of NI is a main safety concern of medical architectural design. Since the 19th century, the ventilation, light, cleaning, noise, and disinfection of building space have been the basic elements that are used to control Nosocomial Infection in hospital buildings [7]. In a Nightingale ward, there is spatial separation between patients and medical staff, a specific distance between patients' beds, air convection by opening windows on both sides of the room, and waste is treated separately [8]. Due to the widespread use of antibiotics, there was not much progress in hospital infection prevention and control in medical building design after this original strategy.

Since the beginning of the 21st century, Nosocomial Infection has been increasingly caused by drug-resistant bacteria in the West. Good environmental design can prevent Nosocomial Infection, so a large number of interdisciplinary studies have been conducted. Previous studies show that medical buildings play an important role in the intervention of Nosocomial Infection, which involves almost the entire cycle of Nosocomial Infection [9–11]. Many practical cases show that good environmental design reduces Nosocomial Infection.

In terms of global COVID-19 prevention and control, many areas must construct Disaster Prevention Hospital Buildings using extremely limited resources. The existing medical buildings must also be transformed in terms of design to address new challenges [12–14]. From the perspective of comprehensive architectural design and public health, there is a relative lack of literature on medical architectural design elements that reduce the risk of Nosocomial Infection. Previous studies mostly focus on hospital management and safety behavior management [15, 16], or pathological analysis from the perspective of epidemiology and contagious infection [17]. At present, no systematic architectural design elements have been proposed, so it is impossible to construct an evaluation framework to determine whether the spatial environment in medical buildings reduces the risk of NI. However, with the continuous improvement in NI prevention and control, the occurrence mechanism for NI is becoming increasingly complex. The effectiveness and efficiency of the spatial environment of medical buildings in terms of NI intervention must be determined to verify whether the required level of prevention and intervention is attainable. In terms of the global spread of COVID-19, it is more necessary to study design decision-making for scenarios using the results of current research, in order to improve the spatial environment in medical buildings and minimize the NI risk in medical buildings.

This study proposes that the construction of this evaluation model requires the embedding of a mutually supportive process for dynamic thinking. A systematic and mutual influence perspective is required to establish and confirm the effect and priority for each evaluation criterion. The contributions of the evaluation study are confined to the ranking and selection of actual cases and provide a basis of reference for further formulation of an improvement strategy [18, 19].

This study determines the key design elements for the spatial environment of medical buildings to reduce the risk of NI and constructs the evaluation criterion framework. In terms of the prevention of the current epidemic, the complex interaction relationship and priority for elements in the evaluation framework are verified. Representative actual medical building cases are used to reduce the risk of NI as the aspiration level for performance evaluation analysis. For each case, a continuous and systematic design strategy is proposed.

This study first summarizes the environmental design elements that affect the NI risk for medical buildings in an analysis of the key literature and uses exploratory element analysis (EFA) to define the core design elements and element categories to build the evaluation framework. DANP is then used to assign Influence Weights (IWs) to the evaluation elements and clarify the Influential Network Relationship Map (INRM) for the evaluation criteria. TOPSIS is used to evaluate and analyze the performance for actual cases. The results of evaluation and analysis show the influence network relationship and priority for elements, and are used to identify the source of key problems and to formulate continuous and systematic environmental transformation design strategies for actual cases.

The main contributions of this study are as follows:

1. The planning and design of spatial environment can affect the behavior of individuals in medical buildings and the activity of infection sources. It can also reduce the risk of Nosocomial Infection in medical buildings. This study uses a review of the relevant literature and analysis of expert interviews to summarize the environmental design criteria for medical buildings that can reduce the risk of Nosocomial Infection. These criteria are the prevention and control measures for Nosocomial Infection.
2. In order to control the risk of Nosocomial Infection, this study confirms the reliability of content and the structure of the environmental design criteria, and constructs a hierarchical evaluation framework to determine the current actual environmental planning and design status of medical buildings.
3. To allow interaction between criteria in the same dimension, this study draws an Influential Network Relationship Map (INRM) and assigns Influence Weights (IWs) to each criterion. In terms of the reconstruction of medical buildings, this allows architects to identify significant problems and the source of actual problems.
4. Using three general hospitals in the Pearl River Delta region of China as actual cases, this study constructs an evaluation framework to evaluate the risk of Nosocomial Infection in actual cases, from the perspective of building environment planning and design. The performance evaluation results and the Influential Network Relationship Map (INRM) and Influence Weights (IWs) are used to study current environmental improvement design strategies for each case from the “source of impact” issues due to limited resources.

## 2. Literature Review

### 2.1. Nosocomial Infection in Medical Buildings

The process of infection is the process by which pathogens enter the human body from the outside, break through its immune system, and cause disease. From the source of infection, infection includes endogenous and exogenous infection. Nosocomial Infection is mainly an exogenous infection [20]. In previous studies, the triangular chain of “infection source environment host” is identified as the main process for Nosocomial Infection. This process involves the source of infection entering the medical building, then entering the human body in a specific way to form an infection, and finally the infected person becomes a carrier and a secondary source of infection (respiratory, body fluid, and environmental pollution).

The main mechanisms for Nosocomial Infection in medical buildings are air, contact, and water transmission [11, 21, 22]. Lenfestey et al. [3] determined that contact transmission is the most common form, accounting for about 80% of all Nosocomial Infections. Air transmission and water transmission are also factors. However, these three forms of transmission often occur intermittently. The main sites of infection are the skin system, the respiratory system, and the digestive system.

Contact transmission can involve direct transmission, indirect transmission, or droplet transmission. Direct transmission occurs through direct contact between individuals, indirect transmission involves an environmental surface as a carrier, and infection occurs when a susceptible person frequently contacts the contaminated surface (with pathogens) [23]. The distance over which droplet transmission operates is very small. It mainly infects the surrounding population through droplets that are discharged from the mouth and nose of the carrier. The liquid particles are large, so they are not suspended in the air for a long time. Research shows that their range is within 1.8 m of the infected person. In contrast to droplet transmission, air transmission occurs after the pathogen particles in the air enter the human body. It may be suspended for a long time and spread with the airflow. Therefore, this route of transmission is the most difficult to prevent, especially in confined spaces. The pathogens for this route of transmission can come from carriers, or the environment, in water, dust, or biological excreta [24]. These pathogens are mainly transmitted by aerosol and dust. The former are generally dry-weather-resistant pathogens, such as measles virus and tuberculosis. Dust transmission is relatively rare, often involves fungal pathogens, and mostly occurs during building renovation.

Although there are only few cases in which Nosocomial Infection is caused by water transmission, pathogens breed easily in water. The ideal temperature is different for different pathogens [25]. The specific mechanisms for water transmission include water contact, drinking, and aerosol infection. The pathogen for this route of transmission travels mainly through daily contact during washing, showering, or drinking, and by aerosol inhalation in the body [26, 27]. These three transmission routes mainly occur in nursing units, wards, emergency halls, and other places. Contact transmission can also occur in infusion centers, and outpatient and laboratory departments. Air transmission can also occur in infection departments and water transmission can occur in public toilets.

### 2.2. The Effect of the Spatial Environment on Nosocomial Infection

The spatial environment in medical buildings is the main carrier of Nosocomial Infection sources. Physical conditions, such as light, temperature, humidity, air flow, and cleanliness, have a significant effect on the activity of pathogens. Previous studies show that ultraviolet light in natural light reduces the survival time for most bacteria and inhibits reproduction [28]. COVID-19 has been the subject of many studies since its outbreak [29, 30]. The effect of environmental temperature and humidity on COVID-19 activity has been studied. Goswami et al. [29] collected daily data for New Coronavirus infection cases and climatic elements in India. Regression analysis shows that the interaction between mean temperature and mean relative humidity has a specific effect on the occurrence of COVID-19. The environmental temperature and humidity in medical buildings can be precisely controlled using effective design measures. Changes in air distribution and composition in hospital air also affect the reproduction and activity of NCV [31]. A clean environmental interface for public buildings is also a basic strategy to reduce the risk of new crown infection at a construction site. Recently, many hotels, schools, and hospitals have also updated their cleaning procedures and now use advanced cleaning technologies to improve disinfection (e.g., electrostatic spray or ultraviolet technology).

The spatial environment is also affected by people's behavior. Streamlining and functional design affect the behavior path and unnecessary contact and crossing can be controlled [8]. This is an effective way to reduce the incidence of Nosocomial Infection. More specifically, the chances of contact with the source of infection can be lessened by affecting individuals' behavior and it is the key element of design to restrict the moves of the carriers and susceptible people. This controls Nosocomial Infections and is a basic principle of modern hospital design. It involves the separation of doctor and patient, the separation of cleaning and contamination, and the separation of internal and external. Controlling the scope of action and protecting the susceptible population can also reduce the risk of exposure to Nosocomial Infection.

## 3. Methodology and Process

**Figure 1:**
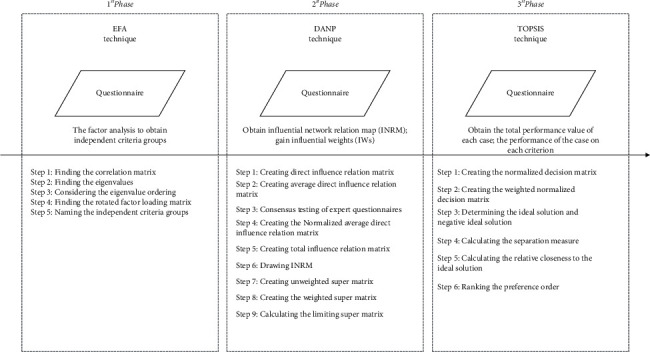
Research procedure for the multiple attribute decision-making model.

The research procedure for this study is shown in Figure 1, which shows the research methods and technical routes that are used in different stages of this study and the corresponding research problems to be solved. The evaluation framework was initially established through a literature review, and an exploratory element analysis (EFA) was used for pretesting to determine whether the structure of the evaluation framework is accurate and whether the evaluation criteria are effective, in order to construct the evaluation framework to determine the risk of Nosocomial Infection in hospital buildings. DANP is used to determine the impact relationship between the evaluation criteria using expert domain knowledge, an Influential Network Relation Map (INRM) is drawn, and Influential Weights (IWs) are assigned to each criterion. In terms of actual case evaluation and analysis, this study uses three hospital buildings in Guangzhou as actual cases to conduct performance evaluation and analysis. Using the performance evaluation questionnaire, TOPSIS is used to determine the current risk of Nosocomial Infection risk for each case and the performance of each case in terms of each criterion. Using the results for each research stage, the influence network relationship and priority of elements are determined to identify the source behind the problems and to formulate continuous and systematic environmental transformation design strategies for these cases.

### 3.1. Exploratory Factor Analysis (EFA)

EFA is used to determine the number of elements that affect variables and to analyze which variables go together [32]. The hypothesis for EFA is that there are *m* common latent elements to be discovered in the data set, and the goal is to identify the smallest number of common elements that account for the correlations [33]. The dependent variables are surface attributes and the underlying structures (factors) are internal attributes [34]. Common elements are those that affect more than one of the surface attributes, and specific elements are those which only affect a particular variable [34]. The main procedure for principal component analysis is described in the following steps when applying exploratory element analysis:

 
**Step (E1)**: determine the correlation matrix **R** or variance–covariance matrix for the objects to be assessed.
 
**Step (E2)**: determine the eigenvalues *λ*_*k*_, *k*=1,2,...,*m* and eigenvectors *β*_*k*_ = [*β*_1*k*_, ..., *β*_*ik*_, ..., *β*_*pk*_] to assess the element loading $a_{\text{i}k}=\sqrt{\lambda_{k}}\beta_{\mathrm{ik}}$ and the number of elements *m*.
 
**Step (E3)**: consider the eigenvalue ordering *λ*_1_ > ... > *λ*_*k*_ > ... > *λ*_*m*_, where *λ*_*m*_ > 1, to determine the number of common elements, and specify the number of common elements to be extracted using a predetermined criterion.
 
**Step (E4)**: according to [35], use the varimax element to determine the rotated element loading matrix, which provides additional insights for the rotation of the element-axis.
 
**Step (E5)**: name the element in terms of the combination of manifest variables.

### 3.2. The DEMATEL-Based ANP (DANP) Method

In previous studies, the analysis methods to determine the relative importance of evaluation criteria for a spatial environment use expert domain knowledge, such as an Analytic Hierarchy Process (AHP) or Analytic Network Process (ANP), and public preference judgment, such as choice-based joint analysis. Many traditional analysis techniques that are used in previous studies must allocate the weight of criteria based on the assumption that the evaluation criteria are independent of each other; so, the influence source of real problems can be neglected when formulating improvement strategies using the analysis results for the current situation evaluation. In order to broaden the assumption of independence, the field of operational research uses the DEMATEL for a multi-criteria decision-making model. In combination with the basic concept of ANP, it is used to train the weight of evaluation indexes by allowing the mutual influence between various evaluation indexes [18, 19, 36]. The main procedure for this method involves the following steps:

**Step (D1)**: establish the direct influence relation matrix **E**. Data are obtained using a questionnaire and the scales for the questionnaire involve an integer score of 0, 1, 2, 3, or 4, where 0 represents absolutely no influence and 4 represents very high influence, according to the natural language element in linguistics. The respondent is assumed to be an expert in the field, and pairwise comparison is used to determine the degree of influence of the element and show the degree to which each element *i* affects each other element *j*. This matrix must be an *n* × *n* nonnegative matrix. The results for *H* experts are used to construct the direct influence relation matrix **E**, as shown in Equation (1), and the direct influence relation matrix for each expert is **E** ^*h*^ = [*e*_*ij*_^*h*^]_*n*×*n*_, *h* = 1,2, ..., *H*.
(1) $$\begin{array}{c} E=\left[\begin{array}{ccccc} e_{11} & \cdots & e_{1j} & \cdots & e_{1n}\\ \vdots & & \vdots & & \vdots \\ e_{i1} & \cdots & e_{ij} & \cdots & e_{in}\\ \vdots & & \vdots & & \vdots \\ e_{n1} & \cdots & e_{nj} & \cdots & e_{nn} \end{array}\right]. \end{array}$$
**Step (D2)**: construct the average direct influence relation matrix **A**. The average scores for the *H* experts **are** *a*_*ij*_ = (1/*H*)∑_*h*=1_^*H*^ *e*_*ij*_^*h*^. The average matrix is called the average direct influence relation matrix **D** and represents the degree of influence that one criterion exerts on another and the degree of influence that the criterion receives from another, as shown in
(2) $$\begin{array}{c} A=\left[\begin{array}{ccccc} a_{11} & \cdots & a_{1j} & \cdots & a_{1n}\\ \vdots & & \vdots & & \vdots \\ a_{i1} & \cdots & a_{ij} & \cdots & a_{in}\\ \vdots & & \vdots & & \vdots \\ a_{n1} & \cdots & a_{nj} & \cdots & a_{nn} \end{array}\right]. \end{array}$$
**Step (D3)**: determine the consensus. The value for consensus is estimated using Equation (3), which represents the degree of consensus for the experts. The statistical threshold for the average gap ratio is 5% and a value less than 5% implies a confidence level of more than 95%, which also represents a stable system. If a system is unstable, the first phase must be implemented again to verify whether data collection is correct and whether the number of experts is sufficient.
(3) $$\begin{array}{c} \mathrm{average}\;\mathrm{gap-ratio}\;\mathrm{in consensus}\;\left(\%\right)=\frac{1}{n\left(n-1\right)}\sum_{i=1}^{n}\sum_{j=1}^{n}\left(\frac{\left|a_{ij}^{H}-a_{ij}^{H-1}\right|}{a_{ij}^{H}}\right)\times 100\%. \end{array}$$
**Step (D4)**: formulate the normalized average direct influence relation matrix **D**. The matrix **D**, which is acquired by normalizing the matrix **A**, is derived from Equations (4) and (5), where all principal diagonal elements are equal to 0:
(4) $$\begin{array}{c} D=b\cdot \mathrm{A,} \end{array}$$
(5) $$\begin{array}{c} b=\min\left\{\frac{1}{\max_{1\leq i\leq n}\sum_{j=1}^{n}a_{ij}},\frac{1}{\max_{1\leq j\leq n}\sum_{i=1}^{n}a_{ij}}\right\}. \end{array}$$
**Step (D5)**: construct the total influence relation matrix **T**. There is a continuous decrease in the indirect effects of problems with the coefficients of the matrix **D**, e.g., **D** ^2^,…, **D** ^*∞*^, and lim_*q*⟶*∞*_ **D** ^*q*^ **=**[0]_*n*×*n*_, for Equation (6), where **I** is a *n* × *n* unit matrix. The total influence relation matrix **T** is a *n* × *n* matrix that is defined by **T** = [*t*_*ij*_]_*n*×*n*_, as shown in Equation (7):
(6) $$\begin{array}{c} \lim_{q⟶\infty }\left(I+D+D^{2}+\ldots +D^{q}\right)\left(\right)={\left(I-D\right)}^{-1}. \end{array}$$
(7) $$\begin{array}{c} T=D{\left(I-D\right)}^{-1}, \end{array}$$
**Step (D6)**: generate the Illustration for the INRM. The total influence relation matrix **T** for the INRM is **calculated** using Equations (8) and (9), which, respectively, generate each row sum and column sum in the matrix **T**.
(8) $$\begin{array}{c} o={\left(o_{i}\right)}_{n\times 1}={\left[\sum_{j=1}^{n}t_{ij}\right]}_{n\times 1}=\left(o_{1},\ldots ,o_{i},\ldots ,o_{n}\right). \end{array}$$
(9) $$\begin{array}{c} r={\left(r_{i}\right)}_{n\times 1}={\left(r_{j}\right)}_{1\times n}^{\prime }={\left[\sum_{i=1}^{n}t_{ij}\right]}_{1\times n}^{\prime }={\left(r_{1},...,r_{j},...,r_{n}\right)}^{\prime }. \end{array}$$
**Step (D7)**: calculate the unweighted supermatrix **W** ^*α*^. Normalize the total influence relation matrix **T**_*C*_ using dimensions (called clusters), as shown in
(10)

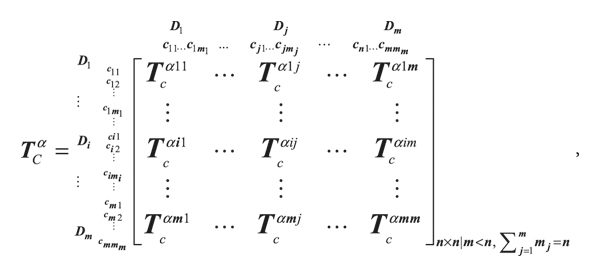

where **T**_*C*_^*α*^ denotes the normalizing total influence relation matrix for criteria by dimensions, and **T**_*c*_^*α*14^ is derived from Equations (11) and (12). Similarly, **T**_*c*_^*αmm*^ can be obtained:
(11) $$\begin{array}{c} t_{i}^{14}=\sum_{j=1}^{m_{4}}t_{ij}^{14},\;\;i=1,\;2,\;\cdots ,\;m_{1}. \end{array}$$
(12) $$\begin{array}{c} T_{c}^{\alpha 14}=\begin{array}{c} c_{11}\\ \vdots \\ c_{1i}\\ \vdots \\ c_{1m_{1}} \end{array}\;\begin{array}{ccccc} c_{41} & \cdots & c_{4}j & \cdots & c_{4m_{4}}\\ \left[\begin{array}{ccccc} t_{\mathrm{11}}^{\mathrm{14}}/t_{1}^{\mathrm{14}} & \cdots & t_{1j}^{14}/t_{1}^{14} & \cdots & t_{1m_{4}}^{14}/t_{1}^{14}\\ \vdots & & \vdots & & \vdots \\ t_{i1}^{\mathrm{14}}/t_{i}^{\mathrm{14}} & \cdots & t_{ij}^{14}/t_{i}^{14} & \cdots & t_{im_{4}}^{14}/t_{i}^{14}\\ \vdots & & \vdots & & \vdots \\ t_{m_{1}^{1}}^{\mathrm{14}}/t_{m_{1}}^{\mathrm{14}} & \cdots & t_{m_{1}j}^{14}/t_{m_{1}}^{14} & \cdots & t_{m_{1}m_{4}}^{14}/t_{m_{1}}^{14} \end{array}\right] \end{array}=\begin{array}{c} \left[\begin{array}{ccccc} t_{11}^{\alpha 14} & \cdots & t_{1j}^{\alpha 14} & \cdots & t_{1m_{4}}^{\alpha 14}\\ \vdots & & \vdots & & \vdots \\ t_{i1}^{\alpha 14} & \cdots & t_{ij}^{\alpha 14} & \cdots & t_{im_{4}}^{\alpha 14}\\ \vdots & & \vdots & & \vdots \\ t_{m_{1}1}^{\alpha 14} & \cdots & t_{m_{1}j}^{\alpha 14} & \cdots & t_{m_{1}m_{4}}^{\alpha 14} \end{array}\right] \end{array}. \end{array}$$
Using pairwise comparisons of the criteria and the basic concept of ANP, the unweighted supermatrix **W** ^*α*^ is obtained by transposing the normalized influence relation matrix **T**_*C*_^*α*^ by dimensions (clusters); that is, **W** ^*α*^ = (**T**_*C*_^*α*^), as shown in
(13)

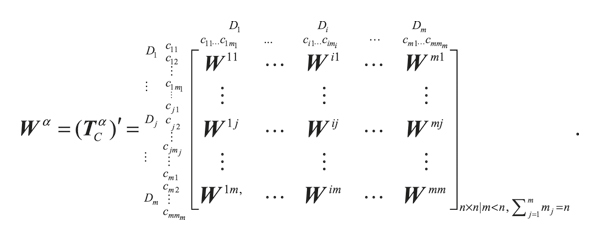

**Step (D8)**: calculate the weighted supermatrix. The normalized total influence-relation matrix of dimension **T**_*D*_^*α*^ is obtained by dividing the total influence-relation matrix **T**_*D*_ by *d*_*i*_ = ∑_*j*=1_^*m*^ *t*_*ij*_, *i* = 1,2, ..., *m*, as shown in Equation (14):
(14) $$\begin{array}{c} {Τ}_{D}^{\alpha }={\left[\begin{array}{ccccc} t_{11}/{\text{d}}_{1}/ & \cdots & t_{1j}/{\text{d}}_{1} & \cdots & t_{1m}/{\text{d}}_{1}\\ \vdots & & \vdots & & \vdots \\ t_{i1}/{\text{d}}_{i} & \cdots & t_{ij}/{\text{d}}_{i} & \cdots & t_{im}/{\text{d}}_{i}\\ \vdots & & \vdots & & \vdots \\ t_{m1}/{\text{d}}_{m} & \cdots & t_{mj}/{\text{d}}_{m} & \cdots & t_{mm}/{\text{d}}_{m} \end{array}\right]}_{m\times m}={\left[\begin{array}{ccccc} t_{11}^{\alpha \;D} & \cdots & t_{1j}^{\alpha \;D} & \cdots & t_{1m}^{\alpha \;D}\\ \vdots & & \vdots & & \vdots \\ t_{i1}^{\alpha \;D} & \cdots & t_{ij}^{\alpha \;D} & \cdots & t_{im}^{\alpha \;D}\\ \vdots & & \vdots & & \vdots \\ t_{m1}^{\alpha \;D} & \cdots & t_{mj}^{\alpha \;D} & \cdots & t_{mm}^{\alpha \;D} \end{array}\right]}_{m\times m}. \end{array}$$
The matrix **T**_*D*_^*α*^, the unweighted supermatrix **W** ^*α*^, and the weighted supermatrix **W** are obtained using Equation (15), where *t*_*ij*_^*α*  *D*^ is a scalar and ∑_*j*=1_^*m*^ *m*_*j*_ = *n*:
(15)

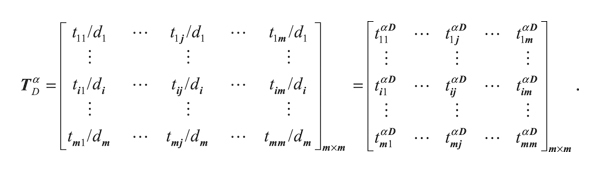

**Step (D9)**: limit the weighted supermatrix. Limit the weighted supermatrix by raising it to the *z*th power until the supermatrix converges and becomes a stable supermatrix. The global priority vectors and the global weight **w** ^*g*^—which are called the IWs of DANP—are obtained, such as lim_*z*⟶*∞*_(**W**)^*z*^, **where** *z* represents any power. By summing the IWs for each criterion in every dimension, the local weight of dimension **w**_*D*_^*l*^ is obtained. The global weight of each criterion is divided by the local weight of its own dimension to yield the local weight of criteria **w**_*c*_^*l*^.

### 3.3. Technique for Order Preference by Similarity to an Ideal Solution (TOPSIS)

TOPSIS is described in [37], with reference to [38]. TOPSIS is a multiple attribute method that is used to identify solutions from a finite set of alternatives. The chosen alternative must be closest to the positive ideal solution and farthest from the negative ideal solution. The procedure for TOPSIS involves a series of steps:

Step**(T1)**: calculate the normalize decision matrix. The normalized value *n*_*ij*_ is calculated using
(16) $$\begin{array}{c} n_{ij}=\frac{x_{ij}}{\sqrt{\sum_{i=1}^{m}x_{ij}^{2}}}\text{ ,}\;i=1,\;...m,\;j=1,...n. \end{array}$$
**Step (T2)**: calculate the weighted normalized decision matrix. The weighted normalized value *v*_*ij*_ is calculated as
(17) $$\begin{array}{c} v_{ij}=w_{ij}n_{ij},\;i=1,\;...m,\;j=1,...n. \end{array}$$
where *w*_*ij*_ is the weight of the *i* − th attribute, and ∑_*i*=1_^*m*^ *w*_*j*_ = 1.
**Step (T3)**: determine the positive ideal and negative ideal solution:
(18) $$\begin{array}{c} A^{+}=\left\{v_{1}^{+},...,v_{n}^{+}\right\}=\left\{\left(\frac{\underset{j}{\max}v_{ij}}{i\in I}\right),\left(\frac{\underset{j}{\min}v_{ij}}{i\in J}\right)\right\},\\ A^{-}=\left\{v_{1}^{-},...,v_{n}^{-}\right\}=\left\{\left(\frac{\underset{j}{\min}v_{ij}}{i\in I}\right),\left(\frac{\underset{j}{\max}v_{ij}}{i\in J}\right)\right\}. \end{array}$$
**where** *I* is associated with a benefit attribute, and *J* is associated with a cost attribute.
**Step (T4)**: calculate the separation from the positive ideal solution as
(19) $$\begin{array}{c} {\text{d}}_{i}^{+}={\left\{\sum_{j=1}^{n}{\left(v_{ij}-v_{j}^{+}\right)}^{2}\right\}}^{1/2},\;i=1,\ldots ,m. \end{array}$$
Similarly, the separation from the negative ideal solution is
(20) $$\begin{array}{c} {\text{d}}_{i}^{-}={\left\{\sum_{j=1}^{n}{\left(v_{ij}-v_{j}^{-}\right)}^{2}\right\}}^{1/2},\;i=1,\ldots ,m. \end{array}$$
**Step (T5)**: calculate the relative proximity to the ideal solution. The relative proximity of the alternative *A*_*i*_ with respect to *A* ^+^ is defined as
(21) $$\begin{array}{c} R_{i}=\frac{{\text{d}}_{i}^{-}}{\left({\text{d}}_{i}^{+}+{\text{d}}_{i}^{-}\right)},\;i=1,...,m. \end{array}$$
Since d_*i*_^−^ ≥ 0 and d_*j*_^+^ ≥ 0, then *R*_*i*_ ∈ [0,1].
**Step (T6)**: rank the preference order for ranking alternatives using this index. Alternatives are ranked in decreasing order.

## 4. Results and Discussion

### 4.1. Environmental Design Elements to Control the Risk of Nosocomial Infection in Medical Buildings

This study focuses on Nosocomial Infection due to risk elements in the environment of medical buildings. The environmental design criteria for medical buildings that reduce the risk of Nosocomial Infection are determined using a review of relevant literature and an analysis of expert interviews. For this study, these guidelines are the prevention and control measures for Nosocomial Infection. The planning and design of a spatial environment can affect the behavior of individuals in medical buildings and the activity of infection sources, and reduce the risk of Nosocomial Infection in medical buildings.

#### 4.1.1. Optimization of Sanitary Ware Layout and Design

Noncompliance with hand hygiene standards is one of the main high-risk behaviors that result in Nosocomial Infection. Many Nosocomial Infections are transmitted through the hands of medical staff [39], so hand hygiene is key to reducing the risk of Nosocomial Infection. Hand contact occurs during almost all medical processes. Practical experience shows that poor arrangement of sanitary ware, insufficient quantities, and poor location in medical buildings reduces compliance with hand hygiene protocols. Hugonnet et al. [40] noted that optimizing the layout of sanitary ware improves compliance with hand hygiene protocols. In a medical building, the number of sanitary stations must fully meet the needs of the individuals that use the building, so the layout of sanitary ware must match the distribution of people and entrances, and waiting areas and other places with a frequent flow of people should have sufficient cleaning stations. Health compliance is related to visual cues. Medical staff often ignore the position of sanitary ware at work so compliance with hand hygiene is increased by the use of induction lamps and significant visual warnings [41]. Noncontact technology also reduces the demand for hand hygiene. Inductive paper drawing, wash basins, and disinfectors reduce the probability of contact, and doors and windows, elevators and other frequently contacted parts can also use inductive technology.

#### 4.1.2. Comfortable and Efficient Public Space

In medical buildings, patients must gather in public spaces for a long time, which increases the risk of contact in the hospital. The indoor environment of medical buildings has a high concentration of pathogens so long stays and frequent transfers significantly increase the risk of Nosocomial Infection. The crowd density in an enclosed space is positively correlated with the infection rate, and poor design can increase the time that patients remain in hospital [42, 43]. Insufficient waiting space, complex and tortuous streamline design, long distances between departments, and poor guide design increase the risk of infection. Azuma et al. [43] noted that in some narrow, crowded, and poorly ventilated indoor environments, aerosol transmission in close contact through some small, atomized particles is combined with respiratory droplets and contact transmission. Although there is little evidence of the impact of aerosol transmission, preventive measures are necessary to control the distribution of individuals in public spaces in medical buildings because COVID-19 has seriously affected global public health, the community, and the social economy.

#### 4.1.3. Control the Crossing and Gathering of Crowd Movement Lines

The design for medical buildings must separate different types of traffic routes to control Nosocomial Infection [44]. The flow lines for common, susceptible, and high-risk groups must be distinguished in terms of the contact risk elements, and the range of movement within the hospital must be controlled [45]. The design for moving lines must use the path-finding characteristics of patients because difficulty in identifying a location is a common reason for unnecessary contact between patients. Specific measures include simplifying the paths, arranging rooms according to patients' path-finding habits, and reducing invalid space transfer [46]. Practical experience shows that a space can be classified according to the risk of Nosocomial Infection and cleanliness. There are ordinary areas, high-risk areas, and buffer areas. Significant buffer areas can be established in different cleanliness conversion areas and materials and colors can be used to emphasize the level of risk.

#### 4.1.4. Appropriate Selection of Environmental Interface Materials

The interior of medical buildings is a closed space, so pathogens can survive in this internal environment for a long time [47]. Some frequently contacted environmental surfaces are significant vectors for Nosocomial Infection. The use of inappropriate environmental interface materials increases the risk of Nosocomial Infection. Carpet allows bacteria to breed and is difficult to clean, and the fabric is easily soaked and can become moldy [48, 49].

Pathogens also adhere to air particles and propagate for a long time, and volatilize into the air to form suspended pathogenic particles [50]. Therefore, environmentally friendly PVC is suited for wall decoration in hospitals. It has unique active antibacterial and self-cleaning characteristics. The surface coating is maintenance-free and surface dust can be directly cleaned using clean water. It has a minimal friction coefficient and experiences minimal dust fouling. Depending on the characteristics of hospital space, new materials, such as bactericidal color latex paint, nano coatings, and antibacterial glass may be appropriate. The selection of materials must be informed by the rationality and practicability of the materials. Decorative materials in toilets must be waterproof and easily cleaned, operating rooms should use antibacterial glass partitions, and the corridors and wards in the hospital should be decorated with bactericidal color emulsion paint.

#### 4.1.5. Higher Proportion of Single Compartments

Nursing units, operating rooms, and wards in medical buildings should achieve better space partition, and the proportion of single rooms should be increased to reduce the probability of contact transmission [51]. Practical experience shows that increasing the proportion of single compartments increases air quality (filtration, ventilation, and air flow control) in medical buildings. Previous studies have shown that under the same environmental conditions, the risk of Nosocomial Infection in a single room is significantly lower than that in a room with multiple occupants or an open ward [52], and the risk of water transmission in the toilet in a single room is also lower, especially in ICU and infection wards. Single room can also become isolation wards in case of an outbreak of an infectious disease. In practice, however, we are often faced with limited space, which can be tackled using the spatial design techniques to dismantle a cluster of units and increase the physical distance between them.

#### 4.1.6. Correct Air Circulation and Purification

Hospital buildings are usually designed to have windows that face the sun and high ceilings because fresh air and good lighting reduce the risk of Nosocomial Infection. Previous studies show that a building's properties, especially the source of ventilated air and the airflow rate, are related to the diversity and composition of indoor bacterial communities [53]. Hobday and Dancer [54] noted that buildings are designed to increase exposure to outdoor air and sunshine to inhibit the survival and transmission of indoor infectious agents. However, many hospitals rely on mechanical ventilation, so air flow and filtering must be designed to prevent Nosocomial Infection. The concentration of pathogens in outdoor natural air is generally lower than that in indoor air, and natural air has a good microbial balance. So, many countries or regions require hospitals to use passive natural ventilation, rather than relying mainly on air conditioning or mechanical ventilation [54]. Previous studies show that opening windows in an ICU significantly reduces the probability of respiratory tract infection. The infection rate for SARS is related to the area of opening windows in a room [55]. Designers should actively use natural ventilation and mechanical ventilation to control indoor air flow and cleanliness, in order to reduce the risk of Nosocomial Infection.

#### 4.1.7. Good Planning of Negative Pressure Isolation Areas

For severe respiratory infectious diseases, such as COVID-19 and SARS, negative pressure wards are used to control Nosocomial Infection [56]. Since the spread of COVID-19, many countries have noted a lack of vacuum isolation rooms, which increases the risk of Nosocomial Infection, aggravates patients' condition, and increases mortality [57]. The isolation area in a hospital has a ventilation system that features negative pressure to prevent infectious diseases from spreading to adjacent rooms or areas. Viruses are isolated and cross-infection is reduced [58]. The air distribution in a negative pressure isolation ward must control the direction of air flow in the ward such that the medical staff are on the windward side of the room and patients are on the leeward side.

#### 4.1.8. Avoid the Accumulation of Water on the Surfaces of an Environment

Water is a breeding environment for the majority of pathogens, and water indoors can volatilize into the air to form pathogenic particles through contact with human skin and respiratory tract infection. Hospital Aspergillus can cause fatal infection in patients with low immune function, so Anaisie et al. [59] conducted a three-year study and determined that air and the hospital's water supply system are the main sources of hospital Aspergillus. Poor environmental design produces spaces that are difficult to clean, and if there is long-term water accumulation, the area can become a breeding ground for infection. These areas include water storage tanks, wash basins, shower heads, and decorative water systems [60].

In order to curb the breeding of pathogens, medical buildings should feature reduced water flow. Water vapor is also a potential source of infection and ventilation, and light controls the propagation of pathogens in water vapor. So, hospital buildings should use natural ventilation and light because ultraviolet light in sunlight inhibits mold and fungal pathogens.

### 4.2. Establish an Evaluation Framework for the Design of Medical Buildings' Spatial Environment to Curb the Risk of Nosocomial Infection

The general qualitative analysis of the relevant literature and expert interviews show that there are eight key environmental design elements. This study uses these environmental design elements to inhibit infection in medical buildings and uses a Likert Five-Point scale questionnaire. Subjects assigned a degree of approval for each item on the basis of actual experience in the prevention of Nosocomial Infection in medical buildings or the design of medical buildings' spatial environment. The scale has five levels: agree very much, agree, neither agree nor disagree, disagree, and disagree very much. The respective scores are 5, 4, 3, 2, and 1. A question that is not answered has a missing value. The subjects of the questionnaire included nurses in the Department of Nosocomial Infection, doctors, and researchers in the field of public health and who design medical buildings' spatial environment. All of the respondents had more than 3 years' work experience and 67 have had a master's degree or higher qualification. Sixty-one were female and 54 were male. A total of 115 questionnaires were distributed for this study and103 were valid.

This study uses EFA to determine the content validity and potential structure of environmental design elements, and then constructs an evaluation framework. Principal component analysis (PCA) and maximum variation (varimax) are used to select a characteristic value greater than 1, and items with elemental loads of less than 0.6 are deleted. Hundred three samples were suitable for element analysis (KMO = 0.729). In terms of reliability, the Cronbach *α* for the eight items is 0.718. The results show that two common elements have eigenvalues greater than 1 and the total cumulative explained variance is 82.431%. These eight evaluation criteria are summarized into two dimensions and the elemental load of each criterion is greater than 0.6. The two dimensions are guiding favorable behavior (*D*_1_) and reducing the source of infection (*D*_2_) (see Table 1). For the guidance of favorable behavior (*D*_1_) dimension, there are four evaluation criteria: the optimization of sanitary ware layout and design (*C*_11_); the control of the intersection and aggregation of crowd moving lines (*C*_12_); a higher proportion of single compartments (*C*_13_); and a comfortable and efficient public space (*C*_14_). These feature good internal consistency (Cronbach *α* = 0.931). There are also four evaluation criteria for reducing the source of infection (*D*_2_): correct air circulation and purification (*C*_21_); selection of appropriate environmental interface materials (*C*_22_); avoiding water accumulation on environmental surfaces (*C*_23_); and good planning of negative pressure isolation areas (*C*_24_). These also feature good internal consistency (Cronbach *α* = 0.887).

### 4.3. Determine the Influential Network Relationship Map (INRM) and Influence Weights (IWs) for the Evaluation Criteria

Exploratory element analysis gives a hierarchical evaluation framework with two dimensions and eight criteria. Weights are assigned to each criterion to allow interaction between the criteria in the same dimension. DANP is used to analyze the evaluation framework, draw the Influential Network Relationship Map (INRM) for each criterion in the same dimension, and assign the Influence Weights (IWs) for each criterion. The subjects for the DANP questionnaire were experts and scholars in the fields of nursing, public health, and medical architectural design. They all have many years of practical and scientific research experience (more than 5 years), and most have PHD degrees (79%). A total of 40 expert questionnaires were distributed, 34 valid questionnaires were returned, and the consistency of expert opinions was verified.

**Figure 2:**
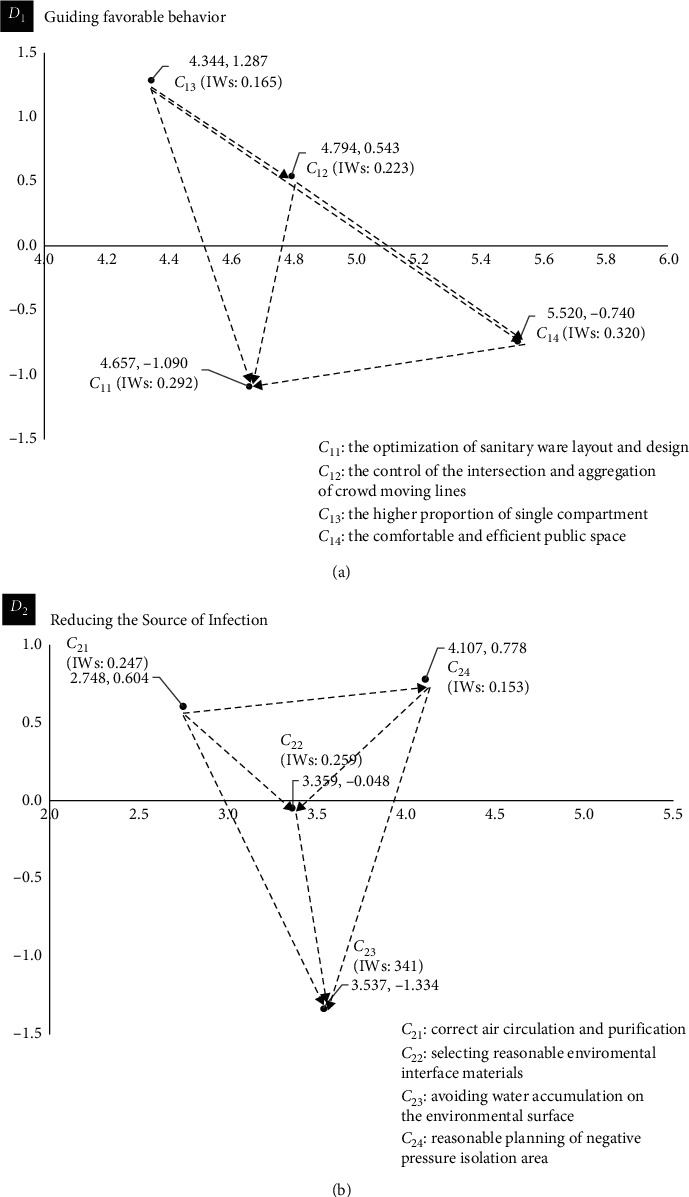
The Influential Network Relationship Map (INRM) for each evaluation criterion in the dimension.

The results show that (as shown in Figure 2) in the *D*_1_ dimension, the most influential criterion is a high proportion of single compartments (*C*_13_), followed by the cross and aggregation of crowd moving lines (*C*_12_), and the weakest criterion is the optimization of sanitary ware layout and design (*C*_11_). In the *D*_2_ dimension, the most influential criterion is the good planning of negative pressure isolation areas (*C*_24_) and the others are: correct air circulation and purification (*C*_21_), appropriate environmental interface materials (*C*_22_), and avoiding water accumulation on environmental surfaces (*C*_23_). Figure 2 shows that for the prevention of Nosocomial Infection in medical buildings, some evaluation criteria have strong Influence Weights (IWs), but their dominant influence on other criteria is weak. These include comfortable and efficient public space in *D*_1_ (*C*_14_) and avoiding water accumulation on environmental surfaces in *D*_2_ (*C*_23_).

### 4.4. Determine the Risk of Nosocomial Infection for Real Cases from the Perspective of Medical Buildings' Spatial Environment Design

#### 4.4.1. Description of Real Cases

COVID-19 has affected many aspects of daily life. Different types of public buildings and open spaces in cities must undergo new inspection protocols to meet requirements for epidemic prevention and control. Medical buildings across China are facing unprecedented challenges. In China, high-quality medical resources are mostly concentrated in the first-tier cities, but even in these cities, there is still a gap between the overall shortage of medical services and the growing demand. The Pearl River Delta region has an advanced manufacturing base and a modern service industry base. The region is one of the main portals for China's foreign exchange and cooperation, and the cities with the highest annual net population inflow are in this region.

Three tertiary hospitals in the Pearl River Delta region are selected as actual cases, with case numbers SYUH, GZMH, and ZCWH. The first two hospitals are located in Guangzhou and the third is in Zhuhai. These three hospitals comprise multiple medical buildings with complex functions. They are also expanding on extremely limited land. They must prevent the current epidemic, but the hospitals are undergoing rapid development.

Case SYUH is in Tianhe District, Guangzhou. At present, the hospital area is roughly divided into three functional areas: the north side is the diagnosis and treatment area; the south side is the living area; and between the diagnosis and treatment area and the living area is the central green space of the hospital. In the medical buildings, the first floor to the fourth floor of the outpatient building, the medical technology complex building, the experimental building, and the inpatient building are connected by a corridor and the underground part of each building is also connected by an underground passage. The main entrance to the north side of the hospital is short of land, crowded, and mixed. The first-floor emergency hall is spacious and bright, and meets the spatial needs of emergency rescue. The emergency area is clearly divided. On weekdays, there are too many patients in the waiting space on each floor of the medical technology complex. There are several nursing units in the medical technology complex and the proportion of single rooms is low. Most wards in the inpatient building have windows that face south and the sanitary ware in the public space is insufficient and the design is old. The negative pressure isolation ward in the hospital was originally an ordinary ward and now uses a high-power exhaust fan for ventilation.

Case GZMH is in Changgang District, Guangzhou City, with a total floor area of 111000 square meters and a building area of 219300 square meters. The hospital area comprises an outpatient area to the north, an emergency area to the south, and an inpatient department in the middle. The fever clinic is located on the first floor of the administration building, adjacent to the inpatient building. The hall in the outpatient building is high, spacious, and bright, and waiting patients and accompanying people gather or stay for a long time in the public space. In contrast, the entrance hall of the emergency building is low and there is a clear moving line. The area of public toilets in the inpatient department and the catering department of the hospital district center is relatively common, and sanitary ware is insufficient. In the inpatient ward of the infectious diseases department, the proportion of single rooms is insufficient (generally 5 people), the indoor air humidity is high, and the ventilation depends on a high-power exhaust fan and natural ventilation.

As the third practical case for this study, ZCWH is in Xiangzhou District of Zhuhai City, adjacent to Gongbei port. The hospital covers an area of 42000 square meters and has a building area of 68000 square meters. The hospital area comprises an outpatient building in the east, a physical examination center building in the west, and an inpatient department in the middle. The emergency area is located on the first floor of the west side of the outpatient building and is connected to the inpatient department by a corridor. There are nursing units on each floor of the outpatient building, and it is not common for people to gather or stay in the waiting area. The first floor of the inpatient department is a registration hall with a high lift and payment hall, and the second floor and above house wards for different departments. Most wards have opening windows to the south. The proportion of single rooms is not high, but a large number of wards are double rooms with an independent bathroom and a public toilet. The negative pressure isolation area is divided based on the current situation of the hospital. The ventilation and purification of indoor air relies on natural ventilation, combined with air conditioning and an exhaust fan.

#### 4.4.2. Evaluating the Risk of Nosocomial Infection for Real Cases

Based on the analysis results of the first two research stages, this study uses TOPSIS to determine the performance of these real cases in terms of building environment planning and design, and determines the risk of Nosocomial Infection for these real cases. A questionnaire (0–10 scale) was distributed to experts who have both architectural design and public health knowledge. An evaluation team of 15 experts from Guangdong, Hong Kong, Macau, and Taiwan was established. These individuals have a master's degree or a higher qualification, have accumulated more than 3 years of working experience, and have participated in scientific research projects that are related to medical architectural design engineering and public health management. The experts in the evaluation team are all familiar with the three real cases, and in the past year, most experts have conducted field surveys in these three hospitals. Some of the experts were hospitalized in the three hospitals and observed and recorded for a long time.

The performance evaluation results for these real cases (Table 2) show that from the perspective of guiding favorable behaviors to curb the risk of Nosocomial Infection, the risk of Nosocomial Infection in the three real cases is ranked from low to high as: ZCWH; GZMH; SYUH. From the perspective of reducing the source of infection to controlling the risk of Nosocomial Infection, the performance ranking for the three real cases is: ZCWH; SYUH; GZMH. The results in Figure 2 show that ZCWH, which has the highest comprehensive performance ranking, achieves high expert scores for the two criteria with the highest influence weight in the *D*_1_ and *D*_2_ dimensions. SYUH has the worst performance in guiding favorable behaviors to curb the risk of Nosocomial Infection. The hospital does not separate different types of traffic routes and indoor crowd crossing and unnecessary gathering is the most serious. Compared with ZCWH, SYUH's registration hall, outpatient waiting area, and other public spaces are not spacious and bright, and the distribution efficiency is low.

GZMH has the worst performance in reducing the source of infection to control the risk of Nosocomial Infection. There is a significant accumulation of water on the surfaces of the hospital environment, the air humidity in the wards is high, and the indoor air cleanliness is low.

#### 4.4.3. Explore the Improvement Strategies for Building Environment Design for the Real Cases to Control the Risk of Nosocomial Infection

Based on the interaction between the evaluation criteria (Figure 2), combined with the performance evaluation results for real cases (Table 2), this study identifies the source of real problems for each case and develops continuous improvement strategies for each case. In order to promote and guide the behaviors that are conducive for restraining the risk of Nosocomial Infection in medical buildings, this study finds that both SYUH and GZMH must focus on existing resources to improve the proportion of single rooms in the hospital. If the space is deconstructed and reorganized, all types of unit compartments can be close to single rooms. It is common to use a flexible partition to split and combine horizontally. If the two cases can split the existing functional groups and design modular standard units, the proportion of single rooms will be increased and the nursing efficiency and adaptability of the building itself will undergo a beneficial functional transformation. If the unit compartments are transformed, the crowd movement line can be indirectly controlled and a one-way streamline and subcenter can be realized. Flexible partitions can be used to create a semi-enclosed group waiting mode, which controls the seat distance and personnel density and improves the comfort and distribution efficiency of the public space.

SYUH and GZMH have poor performance in terms of reducing the source of infection. According to the risk factors for airborne transmission, SYUH and GZMH must modify the design of the hospital air-conditioning and ventilation system to control and reduce the flow direction and concentration of airborne particles. Air must flow in an appropriate direction and from clean areas to semi-polluted areas to buffer area and then to polluted areas. In terms of the overall planning and design of the hospital, the areas where the most vulnerable patients are located (such as operating rooms, transplant facilities, and intensive care units) or the areas for patients with infectious diseases (such as infectious disease rooms or isolation wards), it is necessary to use a more powerful mechanical ventilation system to remove potential pathogenic biological aerosols, in order to reduce the risk of infection transmission.

**Figure 3:**
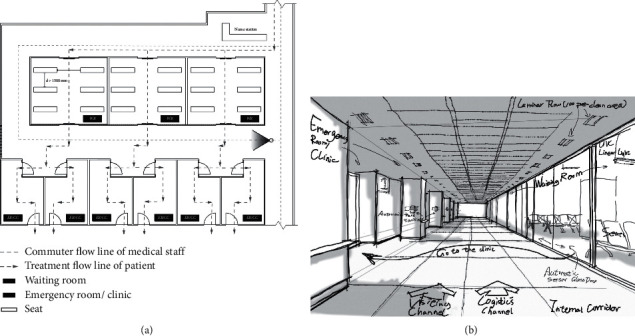
Example of outpatient department design.

ZCWH has the best performance of the three real cases, but Figure 2 and Table 2 show that the hospital could improve its performance in terms of then *C*_13_ and *C*_12_ criteria by optimizing hospital sanitary ware layout and design. This study finds that ZCWH should use data analysis for patients' path-finding characteristics to plan and design a moving line and simplify the path and reduce the unnecessary contact for patients in hospital. The layout for sanitary ware on the moving path of hierarchical planning avoids the waste of resources and allows a hierarchical design for sanitary ware and a guide system. The patients' routing habits mean that the layout of the unit compartments in the hospital can also be adjusted and invalid space transfer of patients in the hospital can be further improved, in order to improve the collection and distribution efficiency of the public spaces. The waiting area and entrance are the key elements for streamline control. It is difficult to identify high-risk groups, so the layout of the waiting area should allow a safe personnel density and control seat spacing. The fully open waiting area is not conducive for the control of Nosocomial Infection and the semi-enclosed group waiting mode can be used (as shown in Figure 3). Flexible partitions could be used to establish an isolation waiting area in pediatrics and obstetrics. When patients enter the hospital, their activities are difficult to control; so, it is necessary to increase control at the entrance and exit, to control the number of entrances and exits and to establish observation and registration facilities.

In terms of reducing the source of infection, ZCWH is significantly worse than the other cases in terms of the most influential *C*_24_ criterion. This hospital should transform some simple negative pressure isolation wards through safe and accessible technical means, in order to reduce the risk of cross-infection. In order to ensure a unidirectional flow of air from clean areas to polluted areas, the air pressure inside the ward must be lower than that outside the room. This is achieved by ensuring reasonable air distribution and keeping the door closed. Although space resources are extremely limited, the hospital should also use three areas and two channels to divide the polluted areas, semi-polluted areas, and clean areas, and clarify the medical channels and patient channels. In the negative pressure isolation ward, transfer windows with interlocking inner and outer windows and doors should be established on the wall of the potential pollution area in the adjacent corridor and the transfer window structure should be closed. The hospital should also plan a buffer area in the isolation area, increase disinfection and sterilization of the environment, and increase air purification. An ultraviolet sterilizer should be used as an auxiliary means to kill pathogens and a chilled beam can also be used as an indoor cooling source to separate the temperature regulation and ventilation functions, in order to reduce airborne infection.

Hospital designers can eliminate infection sources and restrict human behavior by planning the real space in the medical buildings to curb the risk of Nosocomial Infection. The public spaces in medical buildings, where people stay for a long time, are key areas for the prevention of Nosocomial Infection. This is a complex system of a variety of blocks with different functional attributes (outpatient department, inspection and treatment unit). By considering personnel density, residence time, physical environment data, and other relevant information, designers should establish a zoned layout and a design for supporting facilities at the site, to increase the distribution efficiency, safety, and comfort of public spaces. The results in Figure 2 show that designers should coordinate the planning and design of public spaces by considering unit layout and the organization of moving lines. A one-way streamline and a subcenter are ideal for medical buildings. Designers should make full use of space resources and maximize the proportion of single rooms. Flexible partitions can be used to divide polluted areas and semi-polluted areas, and to increase the size of buffer rooms and decontamination dressing rooms. The pedestrian flow line should be one-way and inflow and outflow should be separated. The direction in which doors open and the design of doors can be used to prevent the backflow of pedestrian flow from a polluted area to a clean area. Finally, designers should set corresponding functional attribute units in terms of space division and organization. An independent physiological laboratory and waste treatment system are located in the infectious disease unit to reduce the risk of Nosocomial Infection during waste transfer for on-site pretreatment.

This study finds that reducing indoor water is the most important design criterion for reducing pathogen breeding and Nosocomial Infection risk. The antibacterial and cleaning properties of environmental interface materials are important. Materials that are easy to clean inhibit the sources of infection. Smooth and nonporous materials should be used for frequent contact elements and fabrics, and fiber materials should not be used. Floor materials should be seamless, and stainless steel skirting lines should be used in rooms in which there is a risk of blood and body fluid splashing, and internal corners should be avoided at junctions. The end of water flow should also be reduced. Figure 2 shows that designers should use simple room shapes, control ventilation and light to reduce pathogen reproduction in water vapor, give priority to natural ventilation and light, and use ultraviolet light to inhibit mold and fungal pathogens. In terms of the planning and design of a negative pressure isolation ward, designers should achieve a path for the inflow and outflow of air based on the zoning planning of the front room and ward of the negative pressure isolation ward, introduce sufficient fresh air from outside, and maintain the air exchange rate. Particles to which pathogens attach for transport can be used as tracking materials and the particle removal efficiency can be used as a performance evaluation index for a negative pressure isolation ward to determine the effect of ward size, ventilation times, negative pressure value, and pathogen generation locations, in order to eliminate pathogens as quickly as possible.

## 5. Conclusions

Nosocomial Infection has a negative effect on social health, which directly and indirectly affects all of society. Hospitals are a gathering place for various infection sources, which leads to the risk of Nosocomial Infection. Nosocomial Infection is the early transmission mode for many epidemic infectious diseases, so its prevention and control affects the progress of an epidemic. Good Nosocomial Infection prevention and control mechanisms can prevent the further spread of an epidemic. Drug disinfection has been the main method to keep the hospital environment clean, so the source of infection has become increasingly resistant to drugs and increasingly difficult to control. These elements greatly increase the risk of Nosocomial Infection and the burden of epidemic prevention for the public health system. Nosocomial Infection due to COVID-19 means that hospitals must devise effective methods to prevent and control Nosocomial Infection.

Long-term effective reduction of Nosocomial Infection requires buildings with the correct spatial environment. Using a review of relevant literature, this study determines eight environmental design elements that reduce the risk of Nosocomial Infection: the optimization of sanitary ware layout and design (*C*_11_); the control of the intersection and aggregation of crowd moving lines (*C*_12_); a higher proportion of single compartments (*C*_13_); a comfortable and efficient public space (*C*_14_); correct air circulation and purification (*C*_21_); selection of appropriate environmental interface materials (*C*_22_); avoiding water accumulation on environmental surfaces (*C*_23_); and good planning of negative pressure isolation areas (*C*_24_). In order to transform these design elements into evaluation criteria and construct an evaluation framework, this study uses EFA to determine the effectiveness of the design elements and to clarify the categories (common elements) of the criteria. The evaluation framework that is constructed for this study has two dimensions: guiding favorable behavior (*D*_1_) and reducing the source of infection (*D*_2_). There are four evaluation criteria for each dimension. DANP is used to plot the Influential Network Relationship Map (INRM) and assign Influence Weights (IWs) for each criterion in the same category.

This study uses three general hospitals in the Pearl River Delta of China as real cases and evaluates the risk of Nosocomial Infection for each case from the perspective of the design of medical buildings' spatial environment. An analysis of the performance in these cases and the results in Figure 2 and Table 2 allow designers to determine the most efficient way to transform and update existing medical buildings or construct new medical buildings.

Using the results for real cases, this study embeds a system dynamic, which is an interactive point of view to determine the source of problems, which avoids the waste of resources, and develops a continuous and systematic design strategy for the real case. The main limitation of this study is that the use of EFA to select evaluation criteria and construct an evaluation framework means that the common factors (evaluation dimensions) that are obtained by analysis are independent of each other, so there is no interaction between *D*_1_ and *D*_2_. This study does not use DANP to clarify the INRM and IWs between the evaluation dimensions. The TOPSIS method that is used for this study to assess performance levels for the case studies is an additive method but circumstances are often nonadditive, so future research might use nonadditive methods to assess performance.

## Figures and Tables

**Table 1:** The EFA results for environmental design elements.

(*N* = 103) Evaluation criteria	Evaluation dimension	
	Guiding favorable behavior (*D*_1_)	Reducing the source of infection (*D*_2_)
The optimization of sanitary ware layout and design (*C*_11_)	0.947	
The control of the intersection and aggregation of crowd moving lines (*C*_12_)	0.920	
A higher proportion of single compartments (*C*_13_)	0.889	
A comfortable and efficient public space (*C*_14_)	0.880	
Correct air circulation and purification (*C*_21_)		0.954
Selection of appropriate environmental interface materials (*C*_22_)		0.926
Avoiding water accumulation on environmental surfaces (*C*_23_)		0.904
Good planning of negative pressure isolation areas (*C*_24_)		0.801
Cronbach *α*	0.931	0.887
Cumulative % of variance	82.431%	

**Table 2:** The performance evaluation for the case study using TOPSIS.

The evaluation framework		SYUH	GZMH	ZCWH
Dimensions	Criteria	Influential weights	The normalize value *n*_*ij*_		
Guiding favorable behavior (*D*_1_)	The optimization of sanitary ware layout and design (*C*_11_)	0.292	0.551	0.689	0.470
	The control of the intersection and aggregation of crowd moving lines (*C*_12_)	0.223	0.407	0.598	0.690
	A higher proportion of single compartment (*C*_13_)	0.165	0.516	0.377	0.769
	A comfortable and efficient public space (*C*_14_)	0.320	0.420	0.596	0.685

Reducing the source of infection (*D*_2_)	Correct air circulation and purification (C21)	0.247	0.623	0.430	0.653
	Selection of appropriate environmental interface materials (C22)	0.259	0.465	0.734	0.494
	Avoiding water accumulation on environmental surfaces (C23)	0.341	0.542	0.425	0.725
	Good planning of negative pressure isolation areas (C24)	0.153	0.710	0.522	0.473

Calculating the relative proximity to the ideal solution		*R*_*i*_ ** (*D***_**1**_ **)**	**0.214**	**0.565**	**0.660**
		*R*_*i*_ ** (*D***_**2**_ **)**	**0.433**	**0.369**	**0.619**

## Data Availability

The data that are used to support the findings of this study are included in the article.
